# DNA uptake from a laboratory environment drives unexpected adaptation of a thermophile to a minor medium component

**DOI:** 10.1038/s43705-022-00211-7

**Published:** 2023-01-11

**Authors:** Benjamin Zeldes, Anja Poehlein, Surbhi Jain, Christoph Baum, Rolf Daniel, Volker Müller, Mirko Basen

**Affiliations:** 1 Microbiology, Institute of Biological Sciences, University of Rostock, Rostock, Germany; 2 Genomic and Applied Microbiology & Göttingen Genomics Laboratory, Georg-August University, Göttingen, Germany; 3 Molecular Microbiology & Bioenergetics, Institute of Molecular Biosciences, Goethe University, Frankfurt/Main, Germany

**Keywords:** Bacterial genetics, Molecular evolution

## Abstract

DNA uptake is widespread among microorganisms and considered a strategy for rapid adaptation to new conditions. While both DNA uptake and adaptation are referred to in the context of natural environments, they are often studied in laboratories under defined conditions. For example, a strain of the thermophile *Thermoanaerobacter kivui* had been adapted to growth on high concentrations of carbon monoxide (CO). Unusual phenotypes of the CO-adapted strain prompted us to examine it more closely, revealing a horizontal gene transfer (HGT) event from another thermophile, *Thermoanaerobacter* sp. strain X514, being cultured in the same laboratory. The transferred genes conferred on *T. kivui* the ability to utilize trehalose, a trace component of the yeast-extract added to the media during CO-adaptation. This same HGT event simultaneously deleted a native operon for thiamine biosynthesis, which likely explains why the CO-adapted strain grows poorly without added vitamins. Attempts to replicate this HGT by providing *T. kivui* with genomic DNA from *Thermoanaerobacter* sp. strain X514 revealed that it is easily reproducible in the lab. This subtle form of “genome contamination” is difficult to detect, since the genome remains predominantly *T. kivui*, and no living cells from the original contamination remain. Unexpected HGT between two microorganisms as well as simultaneous adaptation to several conditions may occur often and unrecognized in laboratory environments, requiring caution and careful monitoring of phenotype and genotype of microorganisms that are naturally-competent for DNA uptake.

## Introduction

Adaptive laboratory evolution (ALE) takes advantage of the fact that the stochastic process of random mutation in rapidly replicating cells can lead to elegant solutions to problems that rational designers might never have considered. These experiments are often carried out as part of metabolic engineering efforts to improve host cell tolerance for substrates or products [1]. ALE can be accelerated by the use of chemical mutagens or error-prone polymerases, but given enough time mutations also accumulate simply through repeated passaging of cells on selective conditions. Sometimes the results can be surprising and unexpected, as in the case of *E. coli* gaining the ability to use citrate, present in media as a chelator, as a carbon source during adaptation over 30 000 generations [2].

Evolution and adaptation to new environments is fueled partly by the ability of cells to uptake exogenous DNA (natural competence) [3], which has been proposed i.e. to help thermophiles adapt to extreme environments [4]. Of the few genetic systems for thermophilic microbes, the simplest and most effective rely on natural competence for DNA uptake [5]. For example, screening a broad range of species from the order *Thermoanaerobacterales* revealed that a majority (13 out of 17 strains) exhibited natural competence [6].

*Thermoanaerobacter kivui* is a thermophilic acetogen capable of rapid growth at 65 °C on either simple sugars or H_2_/CO_2_ [7]. When compared to other *Thermoanaerobacter* species, *T. kivui* utilizes a fairly limited set of sugar substrates [8] and it does not produce ethanol; moreover, it is the only acetogenic *Thermoanaerobacter* species [9]. Utilization of disaccharides or longer chain sugars by wild-type *T. kivui* has never been reported. *T. kivui* grows well in a minimal salts medium without vitamin or amino acid sources, although higher cell densities can be reached by addition of yeast extract [7]. The ability to grow in minimal media was essential in development of a genetic system for *T. kivui*, which relies on minimal media (lacking uracil) to provide selective pressure for uptake of a pyrimidine biosynthesis gene in a uracil auxotrophic acceptor strain [10]. The application of the genetic system for *T. kivui* based on natural competence for DNA uptake has made it a good model organism for studying thermophilic acetogenesis [10]. For example, its fructose [10], mannitol [11] and carbon monoxide (CO) metabolism [12], and the role of its hydrogen-dependent carbon dioxide reductase (HDCR) during heterotrophic and autotrophic growth [13] have been elucidated thanks to the availability of genetic tools. Acetogens such as *T. kivui* are prime biocatalysts for the conversion of synthesis gas (syngas, consisting mainly of a mixture of H_2_, CO, and CO_2_) [14, 15]. While *T. kivui* grows on H_2_ + CO_2_, it is not naturally capable of growth on carbon monoxide (CO) [16]. ALE of the wild type (WT) over successive passages on increasing CO concentrations led to an adapted strain capable of growth on 100% CO (CO-strain) [17]. This CO-tolerant strain could serve as a host for conversion of syngas into carbon chemicals [18].

Here, we report that the *T. kivui* CO-strain exhibited robust growth on the disaccharide trehalose, but lost the ability to grow on minimal medium. Genomic and genetic analyses revealed that the strain unexpectedly had taken up DNA from a related *Thermoanaerobacter* species, strain X514. A part of the native thiamine biosynthesis operon was replaced by a trehalose utilization operon via a homologous recombination event, enabling the CO-strain to utilize trehalose, a minor component of the complex media used during adaptation. We reproduced and analyzed this unexpected “laboratory” HGT event; and implications on adaptive laboratory evolution (ALE) are discussed.

## Methods

### Bacterial strains used in this study

A uracil auxotrophic strain TKV002, previously developed from *Thermoanaerobacter kivui* DSM 2030 by deletion of the *pyrE* gene [10], was used as the reference strain, and is referred to here as “wild type (WT)”. A CO-adapted community was previously developed by subjecting cells of DSM 2030 to successive passages at increasing carbon-monoxide concentrations [17], and a uracil auxotroph of a single isolate from this community was generated by deletion of the *pyrE* gene [12]—this CO-adapted ∆*pyrE* strain is referred to throughout the paper as “CO-strain”. Uracil auxotrophic versions of the WT and CO-adapted strains were used with an eye towards future studies, so that both strains could be further genetically manipulated, with the results of this study serving as a baseline. After reviving cells from –70 °C glycerol stocks, both strains were plated on complex agar media, and a single colony of each strain was transferred to liquid medium, serving as inoculum in all experiments described here. *Thermoanaerobacter* sp. strain X514 (ATCC® BAA-938™; strain X514) cells were kindly provided to MB by Prof. M. Adams (Department of Biochemistry and Molecular Biology, University of Georgia, Athens, USA).

### Media components and growth conditions

For routine growth, cells were cultured in complex medium based on the DSMZ 171 medium, with slight modifications introduced during development of the genetic system [10]. Amongst other constituents, the medium contained, as described, 2 g/L yeast extract (Carl Roth GmbH, Karlsruhe, Germany, Art-Nr. 2363, batch #101305488), as well as the vitamin solution from DSMZ medium 141. All media components were mixed, degassed for 20 min with 80% N_2_ / 20% CO_2_, and autoclaved. Defined medium was identical except for the omission of yeast extract, while a no-vitamin (NV-medium) was used in some experiments lacking both yeast extract and the vitamin solution. Glucose and trehalose were prepared as 1 M sterile filtered anaerobic stock solutions and added to media prior to addition of cells. A 500 µg/L thiamine-HCl stock solution (100x relative to the final concentration provided by the vitamin solution) was sterile filtered and made anaerobic, and added to NV-medium at variable concentrations for some experiments. Uracil was added to all media lacking yeast extract (defined and NV) to a final concentration of 0.25 mM from an anoxic stock. Solid media was identical to liquid media except for the addition of 1.5% BD bacto agar (Fischer Scientific, Schwerte, Germany). Plating was carried out as described previously [10]. Colonies were picked with a needle into 10 mL hungate tubes containing 5 mL of the appropriate medium and incubated until growth was visible. For growth experiments, 50 mL serum bottles containing 25 mL of liquid medium were used. Liquid cultures were incubated in a water bath at 66 °C.

### Monitoring growth and metabolites

Growth was determined by optical density (600 nm). Glucose, trehalose, and acetate concentration were determined using HPLC. The column was organic acid resin 300 × 8 mm (CS—Chromatography Service GmbH, Langerwehe, Germany), the system consisted of a Shimadzu (Kyoto, Japan) SIL-20AC autosampler (10 µL sample injection), LC-20AD pump (0.6 mL/min of 5 mM H_2_SO_4_), CTO-20AC column oven (30 °C), and quantitation by comparison to standard curve peak areas determined by RID-10A refractive index detector. Culture samples (0.5 mL) were acidified with 10 µL 50% v/v H_2_SO_4_, centrifuged to remove cells, and stored frozen until measurement.

### SNP sequencing

High molecular weight DNA was isolated from the original CO-adapted community (not ∆*pyrE*) with the MasterPure Complete DNA & RNA Purification kit (Biozym, Hessisch Oldendorf, Germany) as recommended by the manufacturer. Quality of isolated DNA was initially checked by agarose gel electrophoresis and validated with an Agilent Bioanalyzer 2100 using an Agilent DNA 12000 Kit as recommended by the manufacturer (Agilent Technologies, Waldbronn, Germany). Concentration and purity of the isolated DNA was controlled with a Nanodrop ND-1000 (PeqLab Erlangen, Germany) and concentration was determined using the Qubit® dsDNA HS Assay kit as described by the manufacturer (Life Technologies GmbH, Darmstadt, Germany). Illumina shotgun libraries were prepared using the Nextera XT DNA Sample Preparation kit. To assess quality and size of the sequencing libraries, samples were analyzed with an Agilent Bioanalyzer 2100 using an Agilent High Sensitivity DNA Kit as recommended by the manufacturer. Concentrations of the libraries were determined using the Qubit® dsDNA HS Assay kit according to the instructions of the manufacturer. Sequencing was performed on a MiSeq system with the reagent kit v3 with 600 cycles (Illumina, San Diego, CA, USA) as recommended by the manufacturer. For SNP analysis, Illumina raw reads were first quality-trimmed using trimmomatic v0.39 [19] and subsequently mapped against the reference genome of *T. kivui* (CP00917 [20]) using bowtie2 v2.4.1 with end-to-end mode. The pileup file for SNP calling was produced with samtools v1.9 [21] using the mpileup option and subsequently Breseq v0.32.0 [22] was employed for the detection of SNPs and INDELs. As control, Illumina raw reads produced for sequencing of the reference genome [20] were also mapped against itself to detect false positive SNs and INDELs.

### Transformations

*T. kivui* WT cells were pre-cultured overnight on complex media with a mixture of 25 mM glucose and 25 mM trehalose. For transformation, these cells were then passaged into hungate tubes containing 10 mM glucose and 30 mM trehalose, as well as either no DNA (negative control), 5 µg strain X514 genomic DNA, 1 µg of a PCR product amplified from strain X514 gDNA with primers BZq41 and BZ124 (Long PCR), or 1 µg of a PCR product from strain X514 gDNA with primers BZ123 and BZ143 (Short PCR). CO-strain cells without added DNA were treated identically as a positive control. After culturing overnight, the transformed cells were plated in complex agar media containing either glucose (non-selective) or trehalose (selective). Putative transformed colonies were picked off of trehalose plates into trehalose-containing complex medium.

*T. kivui* CO-adapted cells were pre-cultured on NV media with glucose, uracil and 0.1 mg/mL thiamine-HCL (2x compared to DSM141) added. For transformation, these cells were then passaged into hungate tubes with the same media components, as well as either no DNA (negative control), 5 µg *T. kivui* WT gDNA, or 1 µg of a PCR product amplified from *T. kivui* WT gDNA with primers BZ144 and BZ145 (Thi PCR). After culturing overnight, the transformed cells were plated on NV agar containing only glucose and uracil (selective) or NV agar with glucose, uracil, and 10 µg/L thiamine-HCL (non-selective). Wild-type cells without added DNA were treated identically as a positive control. Putative transformed colonies were picked off of NV plates lacking thiamine into liquid NV medium lacking thiamine.

Once putative transformants had grown in selective medium, they were screened with colony PCR. To ensure no cross contamination with strain X514 occurred, primers targeting its *adhE* gene (TETH514_0672) were used. To ensure no cross contamination of WT and CO-strain occurred, part of the *hycB3* gene (TKV_ c19980) was PCR-amplified and sequenced, since the CO-strain has a mutation in this site (identified by SNP sequencing) that allows the two strains to be differentiated. The 16 S rRNA gene of putative transformants was also PCR amplified and sequenced.

### Screening of transformed cells

Routine screening of transformed cells was performed with colony PCR, where 1 µL of a fully grown *T. kivui* culture was used as template. Reference gDNA for PCR controls was extracted from *T. kivui* WT, *T. kivui* CO-strain, and *Thermoanaerobacter* sp. strain X514 cells using a NucleoSpin Microbial DNA kit (Macherey-Nagel, Düren, Germany). As a reference in agarose gel electrophoresis 1 kb DNA Ladder (NEB #N3232, New England Biolabs GmbH, Frankfurt am Main, Germany) was used, for smaller PCR products the 1 kb PLUS DNA Ladder was used (NEB # N3200). Sanger sequencing of PCR products was performed by LGC Genomics GmbH (Berlin, Germany). Screening primers used in this work are listed in Table S1.

### Sequence analysis

Homologous genes described in this work were identified using BLAST (blast.ncbi.nlm.nih.gov; NCBI, Bethesda, MD, USA). GC-rich islands in *T. kivui* were identified using GC-Profile [23] with halting parameter set to 50 and 1000 bp as the minimum length to segment.

## Results

### SNPs in a genome region for trehalose catabolism and thiamine biosynthesis

**Fig. 1:**
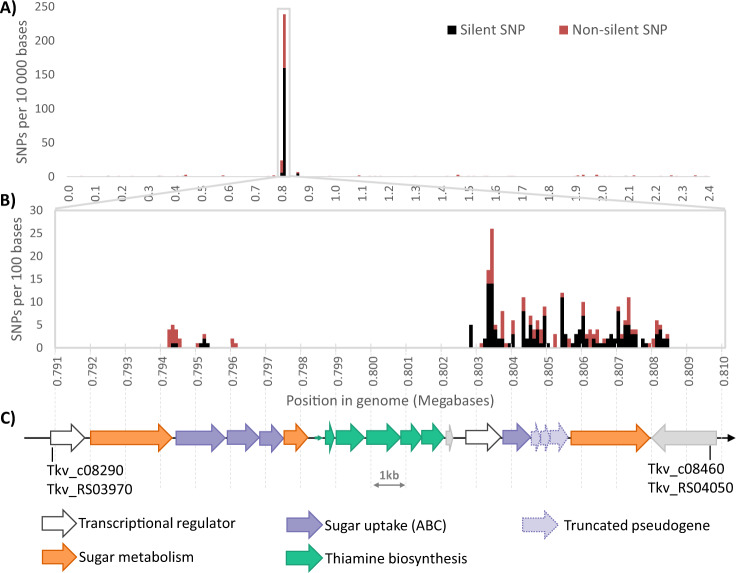
Results of SNP sequencing analysis of the CO-strain of *T. kivui*. Frequency of SNPs in the genome of the CO-strain vs. the wild type: **A** per 10,000 bases in the entire genome, **B** per 100 bases in the region containing the majority of SNPs, black are silent SNPs that do not lead to a change in amino acid sequence, red are non-silent mutations. **C** Genes present in this region of the genome and their probable function.

The genome of the original CO-adapted culture of thermophilic acetogen *T. kivui* [17] was sequenced to identify single-nucleotide polymorphisms (SNPs). Putative mutations conferring CO-tolerance will be described elsewhere. The analysis revealed that over 80% of identified SNPs (263 out of 327 putative mutations) occurred in a single 15 kilobase region of the ~2.4 Megabase genome (Fig. 1A). A closer look revealed two clusters of mutations, separated by a gap of around seven kilobases that contained no mutations (Fig. 1B). Checking the genome annotations for this region reveals that the mutations are in genes annotated as involved in sugar metabolism and sugar uptake via ABC-type transporters, while the mutation-free gap constitutes a thiamine (vitamin B1) synthesis operon (Fig. 1C). The first cluster of mutations occurs near the end of a glycoside hydrolase (TKV_c08300) with 98.6% amino acid identity to a kojibiose phosphorylase from *T. brockii* [24]. This gene is followed by a sugar ABC transporter encoded by TKV_c08310-c08330, the first two genes of which also contain mutations, and a **β**-phosphoglucomutase (TKV_c08340) with no mutations. After the thiamine operon is a second, much higher concentration of mutations in an ABC transport system (TKV_c08410-c08440), although these genes are annotated as truncated pseudo-genes in the reference genome, so likely non-functional (Table S2). The high frequency of mutations continues in the glycoside hydrolase encoded by TKV_c08450, sharing 96% amino acid identity with a trehalose phosphorylase from *Thermoanaerobacterium brockii* Thebr_1548 (**α**, **α**-trehalose phosphorylase, EC. 2.4.1.64) [25]. Assuming the truncated pseudo-genes were once intact, a hypothetical “ancestral” trehalose metabolism for *T. kivui* can be reconstructed, where trehalose enters the cytoplasm un-phosphorylated via the ABC transporter, and is converted into glucose and **β** glucose-1-phosphate by the trehalose phosphorylase. The **β**-phosphoglucomutase may play a role in both kojibiose and trehalose metabolism, converting **β**-glucose-1-phosphate to glucose-6-P for entry into glycolysis (Fig. S1).

The mutation-free area between the two highly mutated regions contains a five-gene thiamine biosynthesis operon, *thiSGHFE* (TKV_c08340-c08390). The first four genes, *thiSGHF*, encode essential enzymes for synthesis of the thiazole moiety of thiamine (the *thiI* gene TKV_c15580 is located elsewhere), and the fifth is one of two copies of the thiamine phosphate sythase (*thiE2* TKV_c08390) gene responsible for combining the thiazole and pyrimidine moieties into thiamine monophosphate [26]. Strain X514 lacks the genes for thiazole biosynthesis, but contains homologues to *T. kivui*’s *thiC* (TKV_c18650) and *thiDEM* (TKV_c03000-3020) genes, which alone are adequate for synthesis of thiamine if thiazole can be salvaged from the medium [27] (Fig. S2).

The unusual concentration of mutations in a small segment of the genome seems to indicate very strong selective pressure in this region. But interestingly, more than 60% of the putative SNP sites in this region (166 of the 263) are silent mutations not resulting in a change at the amino acid level (Fig. 1A, B). The high frequency of SNPs observed here suggests strong selective pressure towards new functions, while the high proportion of silent mutations suggests the opposite: selective pressure to maintain current function [28].

### PCR and Sanger sequencing reveal genomic insertions/deletion

**Fig. 2:**
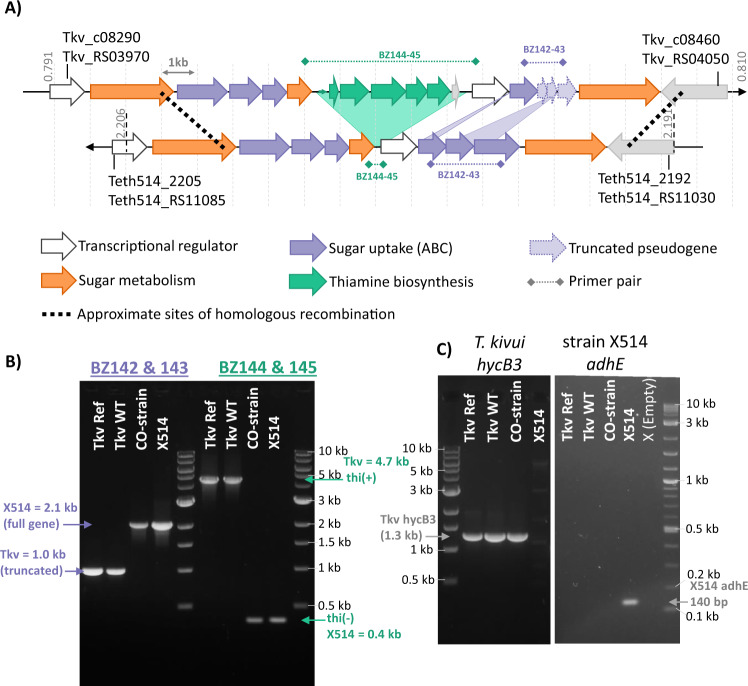
Comparison of highly mutated genome regions of *T. kivui* and *Thermoanaerobacter* sp. strain X514 confirm a horizontal gene-transfer event. **A** Alignment of the *T. kivui* and *Thermoanaerobacter* sp. strain X514 genomes reveals high synteny and sequence homology in the region with the majority of SNPs. **B** Agarose gel electrophoretic separation of PCR products of the ABC-sugar import transporter subunits (purple) and of the thiamine biosynthesis operon (green), obtained by using primer combinations BZ142 and BZ143, or BZ144 and BZ145, respectively. **C** PCR amplification of the *hycB3* gene specific to *T. kivui*, and *adhE* gene specific to strain X514. Genomic DNA purified from: Tkv Ref = *T. kivui* DSM2030; Tkv-WT = *T. kivui* “wild type” (∆*pyrE*) used in this study; CO-strain = *T. kivui* adapted to CO (∆*pyrE*); X514 = *Thermoanaerobacter* sp. strain X514. Marker is NEB 1 kb Ladder, or NEB 1 kb PLUS Ladder (for *adhE* gel).

To confirm the results of SNP analysis, and in an attempt to reconcile these two contradictory lines of thought, portions of the mutated genome region were amplified by PCR and submitted for Sanger sequencing. The resulting PCR product lengths for the CO-strain were not consistent with the published genome (Fig. 2B) as, in addition to the SNPs detected by genome sequencing, several larger insertions and deletions were present. From the PCR, it is apparent that the CO-strain has a roughly 1 kb insertion in the second ABC-transporter cluster (primers BZ142-143) as well as a greater than 4 kb deletion in the thiamine biosynthesis operon (primers BZ144-145) (Fig. 2A).

Interestingly, Sanger sequencing of the anomalous length PCR products revealed perfect sequence agreement to the genome of *Thermoanaerobacter* sp. strain X514, a related strain that differs significantly in its metabolic properties. Strain X514 produces ethanol from glucose by fermentation as well as other alcohols from organic acids [29] but it does not contain the Wood-Ljungdahl pathway for CO_2_ reduction and fixation, and it therefore does not grow on H_2_ + CO_2_ [9]. To rule out the possibility of cross contamination, genes specific to *T. kivui* (*hycB3*) and strain X514 (*adhE*) were amplified, confirming the absence of *adhE* in all *T. kivui* strains and the absence of *hycB3* from strain X514 (Fig. 2C). The 16 S sequence perfectly matched the *T. kivui* 16 S rRNA gene, and contamination was also considered unlikely because the growth capabilities of the CO-strain differ significantly from strain X514 (see below). It is unclear exactly when contamination occurred in the original experiment [17]. The original sample sent for SNP sequencing consisted of a CO-adapted community (generated by repeated passages, but never isolated), from which a single isolate was selected for generation of the ∆*pyrE* CO-strain used here [12]. Since the HGT event is evident in the SNP sequence data, it was clearly present in at least a sub-population of the original adapted community.

An alignment of the two genomes revealed high synteny and sequence homology in this region (Fig. 2A), which would facilitate a HGT event. The most striking differences between the two genomes in this region are the absence of the entire thiamine biosynthesis operon in strain X514 and the absence of ~1 kb from parts of three ABC sugar import genes in *T. kivui* (Fig. 2A and Table S2). These large insertions/deletions do not show up in the SNP sequencing, which relies on mapping reads to the *T. kivui* reference genome, but more subtle differences between the genomes of the two species in the surrounding genes were detected and annotated as single nucleotide mutations. Therefore, rather than hundreds of independent SNP mutations in this region, a single HGT event occurred where the CO-strain of *T. kivui* took up DNA of strain X514 from the laboratory environment and, via homologous recombination, replaced around 15 kilobases of its own genome (containing a thiamine biosynthesis operon) with an 11 kilobase DNA fragment containing a functional ABC sugar import system (Fig. 2). Horizontal gene transfer explains the high frequency of silent SNPs, since the proteins would be evolving independently in both species, but in the context of carrying out similar conserved functions.

Since both strains are studied in our lab, and grow under similar conditions (anaerobic, above 60 °C, in *Thermoanaerobacter* media) we routinely sequence the 16 S rRNA gene of working stocks to exclude the possibility of cross-contamination. A more stringent screen for contamination involves PCR amplification of marker genes specific to suspected contaminants, such as the *adhE* of strain X514 used here (Fig. 2C), but it is important to note that only whole-genome sequencing is adequate to detect the type of “genome contamination” event we describe here. That *T. kivui* can take up and incorporate foreign DNA via natural competence and HGT is well known [10–13, 30], but this is the first report of transformation occurring unintentionally.

### Media components and growth behavior

**Fig. 3:**
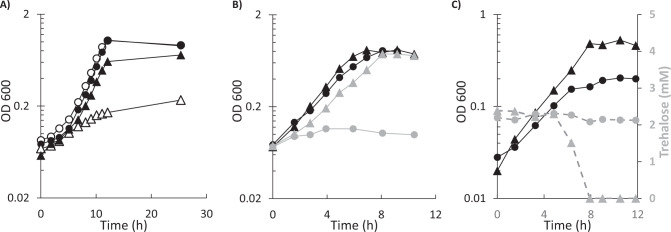
Growth phenotypes of *T. kivui* strains. Growth of *T. kivui* WT (circles) and CO-strain (triangles) on: **A** glucose in NV medium (open shapes) or in NV medium with added thiamine (filled shapes), **B** complex medium with 10 mM glucose (black shapes) or 5 mM trehalose (gray shapes), and **C** 20 g/L yeast extract as source of trehalose—optical density (black shapes), and trehalose concentration (gray shapes with dashed lines). Shown is a single representative run from biological duplicates.

Next, we evaluated the growth of the two WT *Thermoanaerobacter* species and the CO-adapted *T. kivui* strain on trehalose, and determined their requirement for thiamine. As stated in the original isolation report, *T. kivui* grows well in a mineral salts medium without addition of vitamins or yeast extract, so they are not included in the standard medium recommended by DSMZ (medium 171). Yeast extract is naturally rich in thiamine, while supplementation of defined medium with the vitamin mixture results in a final concentration of 5 µg/L thiamine. Strain X514 grew well in *T. kivui* complex media but not in defined media, supporting previous reports finding minimal growth (OD < 0.1) in media without yeast-extract [31]. The CO-adapted strain did not grow in media without vitamins (NV), but addition of 10 µg/L thiamine-HCl from a pure stock recovered normal growth without the need for any of the other vitamins from DSM141 (Fig. 3A). The medium used to adapt *T. kivui* to CO contained both yeast extract and vitamins, and the defined medium used in subsequent experiments also contained the vitamin solution [17], likely explaining why the thiamine requirement of the CO-strain was not noticed before.

Yeast extract is also one of the richest natural sources of trehalose, which can make up between 1 and 10% of yeast cell dry-weight [32]. WT *T. kivui* cells do not grow well on trehalose (Fig. 3B), likely due to the disruptions in the trehalose ABC transporter subunits. In contrast to the WT, the *T. kivui* CO-strain exhibits robust growth on trehalose, nearly as rapid as growth on glucose (Fig. 3B), as does strain X514. To confirm that the CO-strain also exhibited better growth on yeast extract, cells were cultured on NV medium supplemented with 20 g/L yeast extract (ten times the amount in normal complex medium) as the only substrate. WT cells reached a final OD_600_ of 0.201 with a doubling time of 2.2 h, while the CO-strain reached a final OD more than twice as high (0.47) with a slightly faster doubling time of 1.9 h (Fig. 3C). Trehalose concentration in the medium was measured by HPLC before inoculation, revealing that the 20 g/L yeast extract contributed 2.3 ± 0.1 mM trehalose, which would imply that the Roth yeast extract used here is made up of approximately 4% trehalose by dry weight. Within 8 h the CO-strain had completely consumed the trehalose, while the WT strain reduced the trehalose concentration by less than 10% in 24 h. The limited growth observed in the WT was presumably mostly from peptides and other non-trehalose energy sources present in the yeast-extract. When grown on trehalose in complex media (Fig. 3B) the CO-strain was able to generate 5.7 ± 0.2 mM acetate per trehalose (2.9 acetate per glucose), which agrees well with literature values for homoacetogenic utilization of glucose by WT *T. kivui*, in the range of 2.3 to 3.0 acetate per glucose [7, 33].

These results confirm that the genotypes of the respective strains, as revealed by Sanger sequencing, lead to the expected phenotypes. The uptake and integration of a gene region encoding the intact ABC trehalose importer genes from strain X514 confer to the CO-strain the ability to utilize trehalose. The simultaneous deletion of the thiamine biosynthesis operon originally present adjacent to these genes also converts the CO-strain into a thiamine auxotroph. Presumably this gene transfer was facilitated by strong selective pressure, since once other components of the complex media had been consumed, residual trehalose would have represented a promising additional energy source for cells that initially could only poorly utilize CO. To confirm that this genotype could result from an HGT event, we attempted to transform *T. kivui* WT cells with genomic DNA from strain X514.

### Replicating the HGT by transformation with genomic DNA

**Fig. 4:**
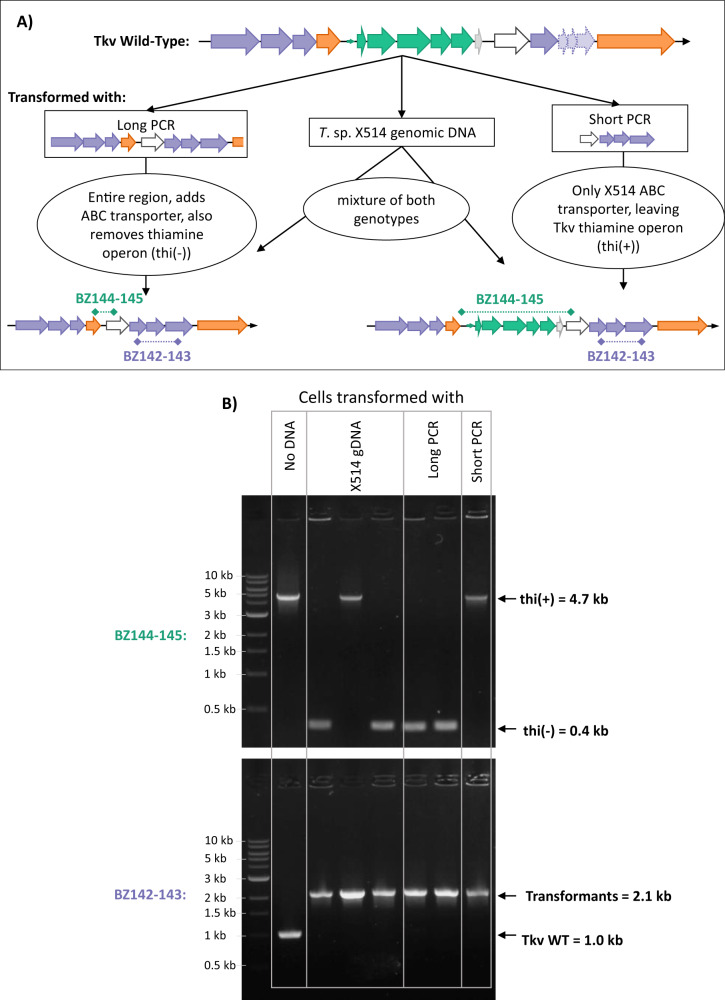
Reproduction of the HGT by transforming *T. kivui* with *Thermoanaerobacter* sp. X514 gDNA, or PCR products of the trehalose region. **A** The transformation scheme displaying original genomic DNA region, the PCR products used, and resulting transformants. **B** PCR screening transformed colonies picked off of selective (trehalose containing) agar plates. The upper gel screens for the presence (4.7 kb) or absence (0.4 kb) of the native *T. kivui* thiamine operon with primers BZ144 and BZ145, while the lower gel screens for presence of the full length X514 trehalose genes (2.1 kb) or truncated *T. kivui* genes (1.0 kb) with primers BZ142 and BZ143. Marker is NEB 1 kb Ladder.

Due to the natural competence of *T. kivui* [10], strain X514 gDNA was simply added to media prior to inoculating a culture of WT *T. kivui* and incubating overnight in glucose media supplemented with trehalose to encourage uptake, since HGT is expected to be a rare event. This was followed by plating on selective agar plates containing only trehalose. Colonies only formed from cultures incubated with added DNA. Transformation with two different PCR products was also successful, both with a short PCR product of 4 kb (Short PCR) containing only the ABC transporter genes from strain X514, as well as with a longer 9 kb product (Long PCR) roughly matching the size of the full HGT region originally identified in the CO-strain (Fig. 4A). Subsequent PCR screening confirmed uptake of the intact ABC transporter genes in all transformed colonies, but colonies transformed with the Short PCR product retained the *T. kivui* WT thiamine biosynthesis operon (*thi*(+)), while those transformed with Long PCR did not (*thi*(-)), and transformation with gDNA resulted in a mixture of *thi*(+) and *thi*(-) colonies (Fig. 4B). Similar results were achieved by transforming the CO-strain with either *T. kivui* WT gDNA or a PCR product of the thiamine operon, and selecting on plates lacking thiamine. Only transformed CO-strain cells formed colonies on the selective plates, but all colonies picked from these plates showed mixed genotypes, suggesting that untransformed cells were cross-fed by thiamine produced by transformants (Fig. S3).

This is the first report of successfully transforming *T. kivui* with genomic DNA, although transformation with both integrating and replicating plasmids has already been demonstrated [10]. Transformation efficiency cannot be accurately determined with this method because transformation occurs concurrently with cell growth, but a rough estimate of relative efficiencies is evident from the number of positive colonies counted on plates. Transformation with gDNA and Long PCR resulted in similar numbers of colonies per ug of the Trehalose-ABC gene operon, while Short PCR was at least two orders of magnitude lower (100x fewer colonies), likely due to a short upstream flanking region of only 100 bp, while all other flanking regions were at least 1000 bp long.

Despite the low transformation efficiency, the fact that integration of the Short PCR product was sufficient to confer growth on trehalose indicates that the first ABC gene operon containing large numbers of SNPs (TKV_c08310-330, Fig. 1) is not responsible for trehalose uptake, since this region was not included in Short PCR. These genes may be responsible for growth on kojibiose (Fig. S1), since *T. kivui* cannot grow on any oligosaccharide tested so far, and kojibiose is an obscure sugar that is difficult to source in pure form. It is found in trace amounts in honey and some fermented products [24], so its biological relevance to *T. kivui* is unclear.

The ease with which transformed *T. kivui* cells containing the full ABC trehalose-import subunits could be isolated on trehalose-containing agar plates (Fig. 4) suggests that the inability to utilize trehalose acted as a strong selective pressure in trehalose-rich environments. Considering that the original HGT event leading to the CO-strain occurred in supposed mono-cultures in a laboratory setting, it is logical to assume that prior to its isolation, *T. kivui*’s genome regularly incorporated DNA from the many diverse species present in its natural environment of Lake Kivu. Therefore, we undertook a closer analysis of its genome to identify potential recent HGTs.

### Evidence of other HGT events in the *T. kivui* genome

The wild type *T. kivui* genome has a relatively low GC content of 35% when averaged across the entire genome. However, an analysis with GC profile reveals three GC-rich islands with greater than 50% GC content (Fig. S4). The first island contains genes encoding ribosomal RNA. Thermophiles are known to have higher GC-content in their 16 S rRNA genes, possibly to stabilize base-paring in the double-stranded regions at high temperatures [34]. The next two islands each contain a gene annotated as aerobic B12 biosynthesis gene *cobN*, as well as ABC-transport components, some annotated as nickel transporters. These two islands are also identified by IslandViewer [35] on the basis of codon usage bias. A BLAST search reveals that no other *Thermoanaerobacter* species contains homologues to these regions, and in fact the only results (nucleotide sequence identity 84–89%) are to genes from two moderately thermophilic sulfate-reducing bacteria *Desulfotomaculum kuznetsovii* [36] and *Candidatus Desulforudis audaxviator* [37]. Both species were isolated from environments between 2000 and 3000 m below the surface. Lake Kivu is unusually deep, approaching 500 m in places [38], so it is not surprising that *T. kivui* encountered relatives of these deep subsurface bacteria, or at least their DNA, in its natural environment prior to isolation. The exact functions of the genes in these clusters remain unclear, since the presence of *cobN*, typically considered part of the aerobic B12 biosynthesis pathway, was surprising in the obligately anaerobic *Desulfotomaculum kuznetsovii* [36]. The *cobN* gene was also identified as a likely instance of horizontal gene transfer from an archaeon in *Candidatus Desulforudis audaxviator* [39].

The importance of HGT in microbial evolution remains controversial, but in certain circumstances it leads to much more rapid development of new capabilities than gradual random mutation [40]. In this context, it is interesting that *T. kivui*, despite sharing high 16 S sequence similarity to other *Thermoanaerobacter* species, is the only acetogenic isolate of the genus so far. This is in contrast to another thermophilic acetogenic genus *Moorella*, for which multiple closely related acetogenic strains have been isolated independently [41]. Since most acetogens encode the genes of the Wood-Ljungdahl pathway in a single large gene cluster, and *T. kivui*’s WLP genes are even more concentrated than most [42], it seems plausible that its acetogenic capabilities could result from an HGT event. The original environmental samples were stored at room temperature for 5 years before *T. kivui* was isolated [7], giving plenty of time for HGT to occur between species present in the samples.

## Discussion

Attempts at adaptive laboratory evolution can have unintended results when cells adapt to alternate selective pressures not anticipated by the researchers. One well known example of unintended selective pressure during ALE concerns the use of chemostats. While theoretically superior to batch culture due to their ability to maintain constant environmental conditions and achieve higher mutation rates by keeping cells in exponential growth indefinitely, chemostat ALE consistently selects for mutations related to cell aggregation or adhesion, regardless of the selective pressure being applied during a specific experiment [43]. These aggregating or adhering cells achieve longer residence times in the chemostat, giving them a competitive advantage over planktonic cells regardless of how well they have adapted to the other conditions in the experiment [44]. Analogous to this universal tendency of cells adapted in chemostats, the CO-adapted strain described here demonstrates that ALE performed on complex media bears the risk of adapting cells to unknown or unanticipated components of the media (in this case, trehalose), in addition to the intended selective pressure (growth on CO).

While the ability to grow on trehalose represents an unintended gain-of-function during an ALE experiment, the loss of thiamine biosynthesis is an unintended loss-of-function, made possible by the fact that the laboratory environment provided no selective pressure to maintain thiamine biosynthesis. An extreme case of loss-of-function mutation during ALE comes from the Long Term Evolution Experiment in *E. coli* (citrate metabolism from the same experiment was mentioned earlier in the context of gain-of-function [2]). After 20,000 generations adapting to growth in batch culture on a minimal glucose medium cells do indeed grow considerably better than the ancestral strain, but tests in environments other than the one used for ALE revealed that the adapted cells had lost the ability to utilize a wide variety of non-glucose substrates [45]. The Long Term Evolution Experiment succeeded in turning *E. coli*, a metabolic generalist [46], into a glucose specialist. Similarly, ALE of *T. kivui* on rich media appears to have begun the process of converting this organism, normally capable of synthesizing all essential vitamins for itself, into one that relies on vitamin uptake from the medium.

The principle of ALE was entirely successful in adapting *T. kivui* to a new growth substrate it was previously incapable of utilizing (CO) [17], confirming ALE as a powerful tool of industrial microbiology alongside more targeted methods such as metabolic engineering and synthetic biology. However, the results reported here should serve as a reminder that careful attention must be paid to the selective pressures present during ALE experiments. The presence of an unforeseen selective pressure can lead to surprising adaptations, while the absence of others can lead to the unexpected loss of desirable capabilities present in the original strain. This example provides an even stronger warning of the possibility of contamination by DNA, meaning that even when utmost care is taken to detect contamination by living cells, incorporation of foreign DNA cannot be detected by anything short of whole genome sequencing. In this case only two species were involved, but the enrichment cultures routinely used to isolate new species out of environmental samples provide the perfect combination of ALE (enrichment conditions) and potential for HGT between the diverse species. It seems possible that enrichments could select for and then lead to isolates with novel hybrid genomes not present in the original environmental sample. In our case this event has proved fortuitous, as it provided a fascinating case-study of natural transformation in *T. kivui*, allowed us to study its vitamin and sugar metabolisms, and revealed important metabolic differences between *T. kivui* and other *Thermoanaerobacter* species.

## Supplementary information

Supplementary Material

## Data Availability

The SNP-sequencing generated during the current study is available in the Sequence Read Archive (SRA), https://www.ncbi.nlm.nih.gov/sra/ (run accession SRR21997958).
