# Supplementary material for: DNA uptake from a laboratory environment drives unexpected adaptation of a thermophile to a minor medium component
Source: ISME Commun. 2023 Jan 11;3:2. doi: 10.1038/s43705-022-00211-7 (PMC9834392; doi:10.1038/s43705-022-00211-7)

**Table S1:** Primers used in this study

| Name | Binds<br>K = *T. kivui*<br>X= strain X514 | Purpose | Sequence |
|---|---|---|---|
| **Cloning** | | | |
| BZq41 | X, K | Long PCR | ATGGAGATGCTTTTGCTGCT |
| BZ124 | X (96%), K | ******single base mismatch to X514* | CGTTATTGTC**G***ACTAATGCCGTATAC |
| BZ123 | X, K | Short PCR | CAATAGACGATGTAGCAAAATTAGCAG |
| BZ143 | X, K | | CTACAAGAACGTTTGCAGTGTAC |
| BZ144 | X, K | PCR thiamine | CATGGAAAACCTGACCCTGAG |
| BZ145 | X, K | | CTGCTAATTTTGCTACATCGTCTATTG |
| **Screening** | | | |
| BZ142 | X, K | Amplify Trehalose insert region | CTTATGGTTTCTGTAGCGGATATGTG |
| BZ143 | X, K | X = 2.1 kb (+tre), K=1.0 kb (-tre) | CTACAAGAACGTTTGCAGTGTAC |
| BZ144 | X, K | Amplify thiamine operon region. | CATGGAAAACCTGACCCTGAG |
| BZ145 | X, K | X = 0.4 kb (-thi), K=4.7 kb (+thi) | CTGCTAATTTTGCTACATCGTCTATTG |
| Eubac fwd | X, K | Amplify 16S gene | GAGTTTGATCCTGGCTCAG |
| Eubac rev | X, K | X= 1.5 kb, K = 1.6 kb | AGAAAGGAGGTGATCCAGCC |
| BZ133 | K | Amplify part of *HycB3* gene | GGATACAGTACCAACCGTCG |
| BZq56 | K | K = 1.3 kb | GCATATGTGATGAAGGGGCTGA |
| BZq71 | X | Amplify part of *adhE* gene | TGGCATCAGACTATACAGACGC |
| BZq72 | X | (Teth514_0672) X= 140 bp | GCCATTCCAGCTATGCATGAAG |

**Table S2**: **Annotations and arrangement of *T.* sp. X514 and *T. kivui* genes in the region of the HGT**

Color coding is the same as Fig. 1. Protein homology results of pBLAST search of *T. kivui* proteins using the corresponding Strain X514 protein amino acid sequence as query.

| | | | | | | | | | | **Homology to *T. kivui* protein** | |
|---|---|---|---|---|---|---|---|---|---|---|---|
| **ORF TKV_** | **Product** | **Start** | **End** | **Length** | **ORF Teth514** | **Product** | **Start** | **End** | **Length** | **% Cov.** | **%ID** |
| **c08290** | **LacI family transcriptional regulator** | 790922 | 791932 | 1010 | **_2203** | **LacI family transcriptional regulator** | 2205689 | 2206699 | 1010 | 100 | 100.0 |
| **c08300** | **glycoside hydrolase family 65 protein** | 791998 | 794325 | 2327 | **_2202** | **glycoside hydrolase family 65 protein** | 2203296 | 2205623 | 2327 | 100 | 97.7 |
| **c08310** | **ABC transporter substrate-binding protein** | 794470 | 795732 | 1262 | **_2201** | **ABC transporter substrate-binding protein** | 2201890 | 2203152 | 1262 | 100 | 99.3 |
| **c08320** | **sugar ABC transporter permease** | 795808 | 796704 | 896 | **_2200** | **sugar ABC transporter permease** | 2200915 | 2201814 | 899 | 100 | 99.0 |
| **c08330** | **carbohydrate ABC transporter permease** | 796718 | 797527 | 809 | **_2199** | **carbohydrate ABC transporter permease** | 2200092 | 2200901 | 809 | 100 | 100.0 |
| **c08340** | **beta-phosphoglucomutase** | 797549 | 798196 | 647 | **_2198** | **beta-phosphoglucomutase** | 2199423 | 2200070 | 647 | 100 | 100.0 |
| **NA** | **TPP riboswitch** | 798447 | 798575 | 128 | | | | | | | |
| **c08350** | **sulfur carrier protein ThiS** | 798707 | 798898 | 191 | | | | | | | |
| **c08360** | **thiazole synthase** | 798963 | 799727 | 764 | | | | | | | |
| **c08370** | **2-iminoacetate synthase ThiH** | 799760 | 800872 | 1112 | | | | | | | |
| **c08380** | **sulfur carrier protein ThiS adenylyltransferase ThiF** | 800869 | 801480 | 611 | | | | | | | |
| **c08390** | **thiamine phosphate synthase** | 801461 | 802126 | 665 | | | | | | | |
| | hypothetical protein (TKV_RS13000) | 802150 | 802296 | 146 | | | | | | | |
| **c08400** | **LacI family transcriptional regulator** | 802666 | 803679 | 1013 | **_2197** | **LacI family transcriptional regulator** | 2198288 | 2199301 | 1013 | 100 | 94.4 |
| **c08410** | **sugar ABC transporter permease** | 803789 | 804499 | 710 | **_2196** | **sugar ABC transporter permease** | 2197338 | 2198195 | 857 | 82 | 97.0 |
| **c08420** | **hypothetical protein** | 804499 | 804708 | 209 | **_2195** | **carbohydrate ABC transporter permease** | 2196508 | 2197338 | 830 | 32 | 95.6 |
| **c08430** | **hypothetical protein** | 804722 | 805009 | 287 | | | | | | | |
| **c08440** | **extracellular solute-binding protein (pseudo gene)** | 805002 | 805538 | 536 | **_2194** | **extracellular solute-binding protein** | 2195193 | 2196437 | 1244 | 45 | 93.6 |
| **c08450** | **glycoside hydrolase family 65 protein** | 805618 | 807960 | 2342 | **_2193** | **glycoside hydrolase family 65 protein** | 2192773 | 2195115 | 2342 | 100 | 96.4 |
| c08460 | S8 family serine peptidase | 808002 | 809732 | 1730 | **_2192** | S8 family serine peptidase | 2191002 | 2192732 | 1730 | 100 | 93.8 |

**Figure S1**: **Hypothetical ancestral pathway for trehalose uptake in *T. kivui***
Enzymes in *T. kivui* homologous to trehalose and kojibiose uptake genes in other *Thermoanaerobacter* sp. Color coding as in Fig. 1 and Table S1.

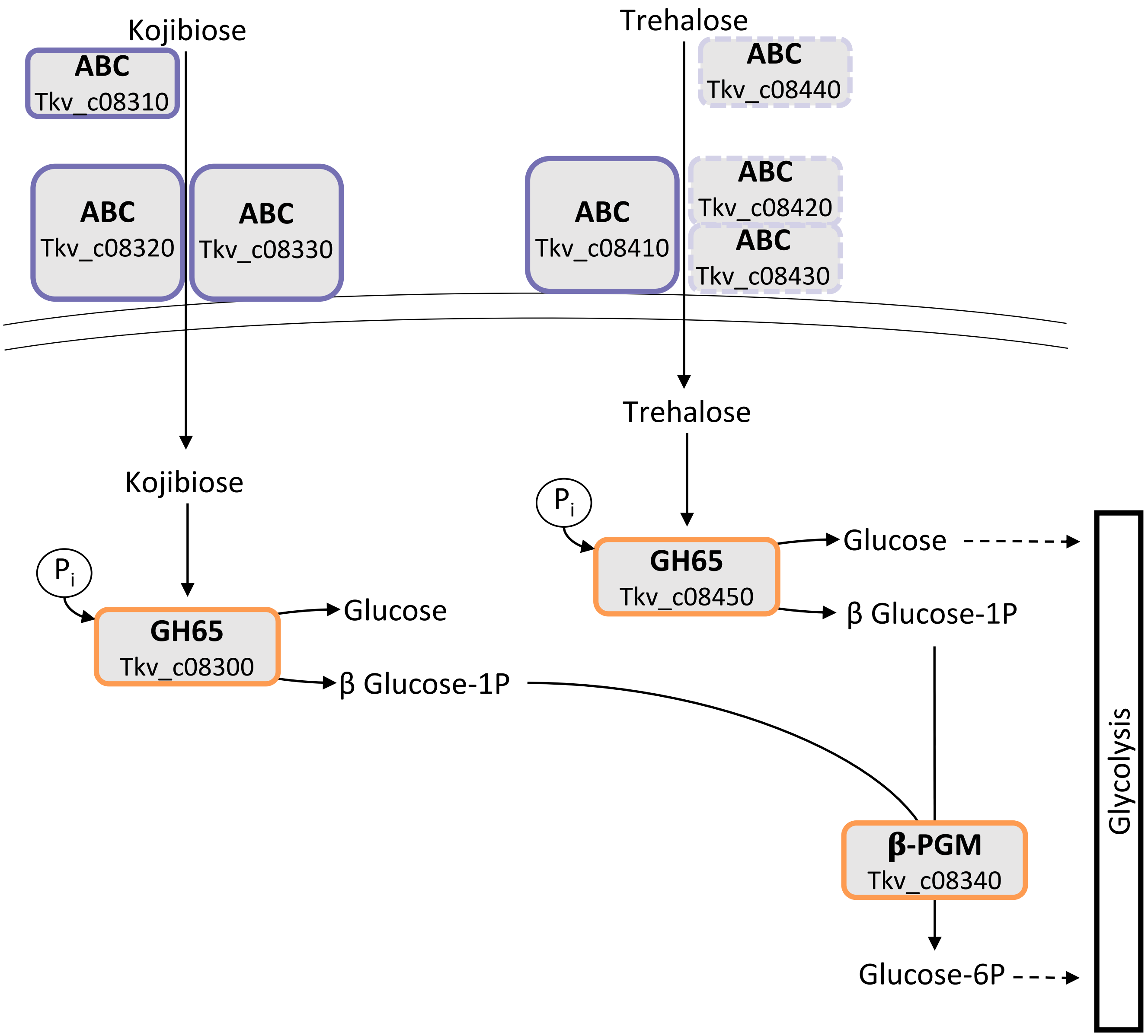

**Figure S2: Proposed Thiamine biosynthesis genes in *T. kivui***

Genes outlined in **green** are from the thiamine operon missing in the CO-strain.

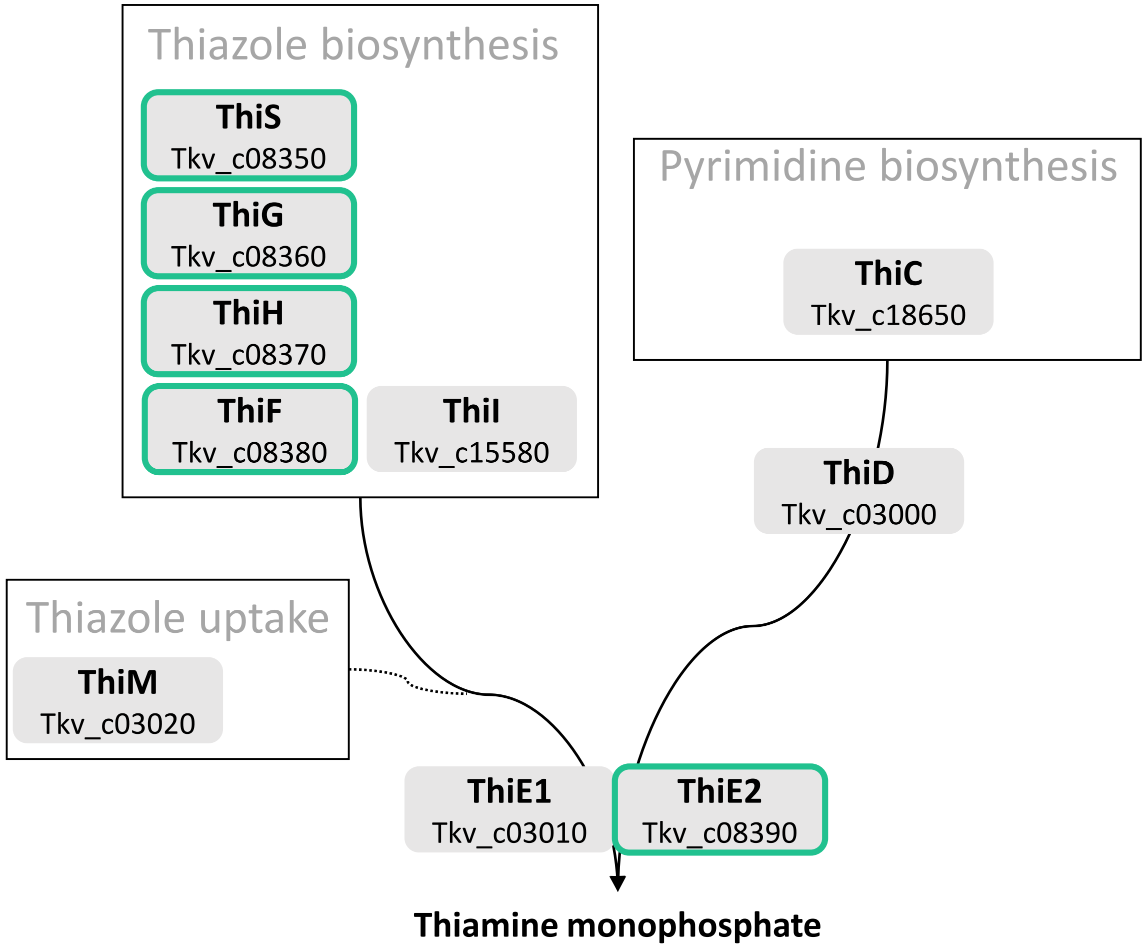

**Figure S3**: ***T. kivui* CO-strain transformed with *T. kivui* WT gDNA or PCR product containing the thiamine biosynthesis genes.**

(A) The transformation scheme displaying original genomic DNA region, the PCR product used, and resulting transformants. (B) PCR screening transformed colonies picked off of selective (NV medium) agar plates. The upper gel screens for presence of the full length X514 trehalose genes (2.1 kb) or truncated *T. kivui* genes (1.0 kb) with primers BZ142 and BZ143, while the lower gel screens for the presence (4.7 kb) or absence (0.4 kb) of the native *T. kivui* thiamine operon with primers BZ144 and BZ145. Marker is NEB 1 kb Ladder.

**A)**

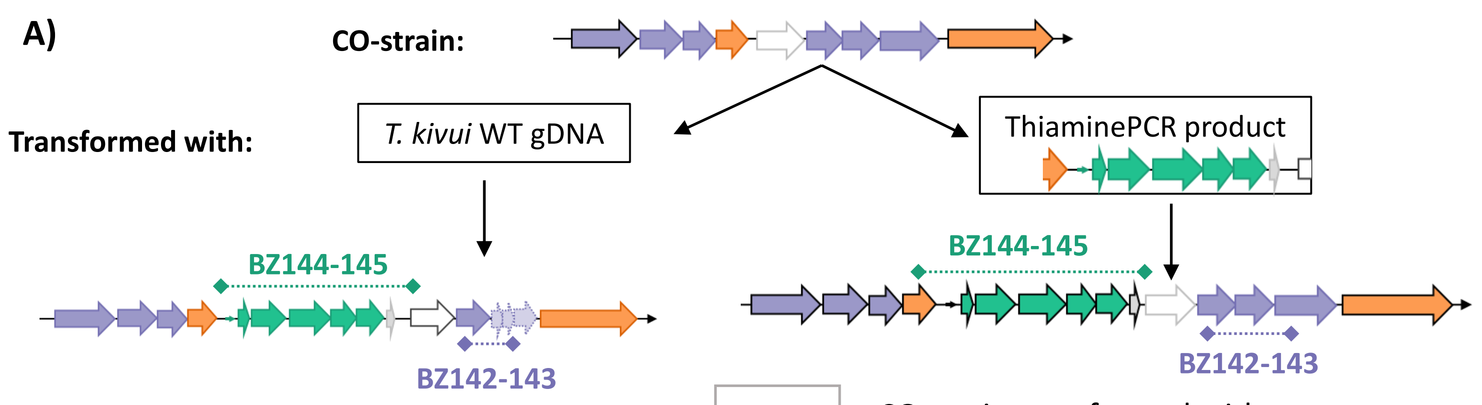


**B)**

Trehalose region (BZ142-143)

Cells transformed with PCR product retain the CO strain genotype with full length Trehalose genes, but cells transformed with gDNA mostly also pick up the Tkv-genotype lacking the Trehalose genes. (the one exception lacks the Thiamine operon - untransformed cross-feeder?)

Thiamine region (BZ144-145)

All but one colony picked off selective plates (lacking thiamine) contain thiamine genes from Tkv-WT.

Original CO-strain genotype lacking thiamine operon is also present in most samples (cross-feeding from the transformed colony?)

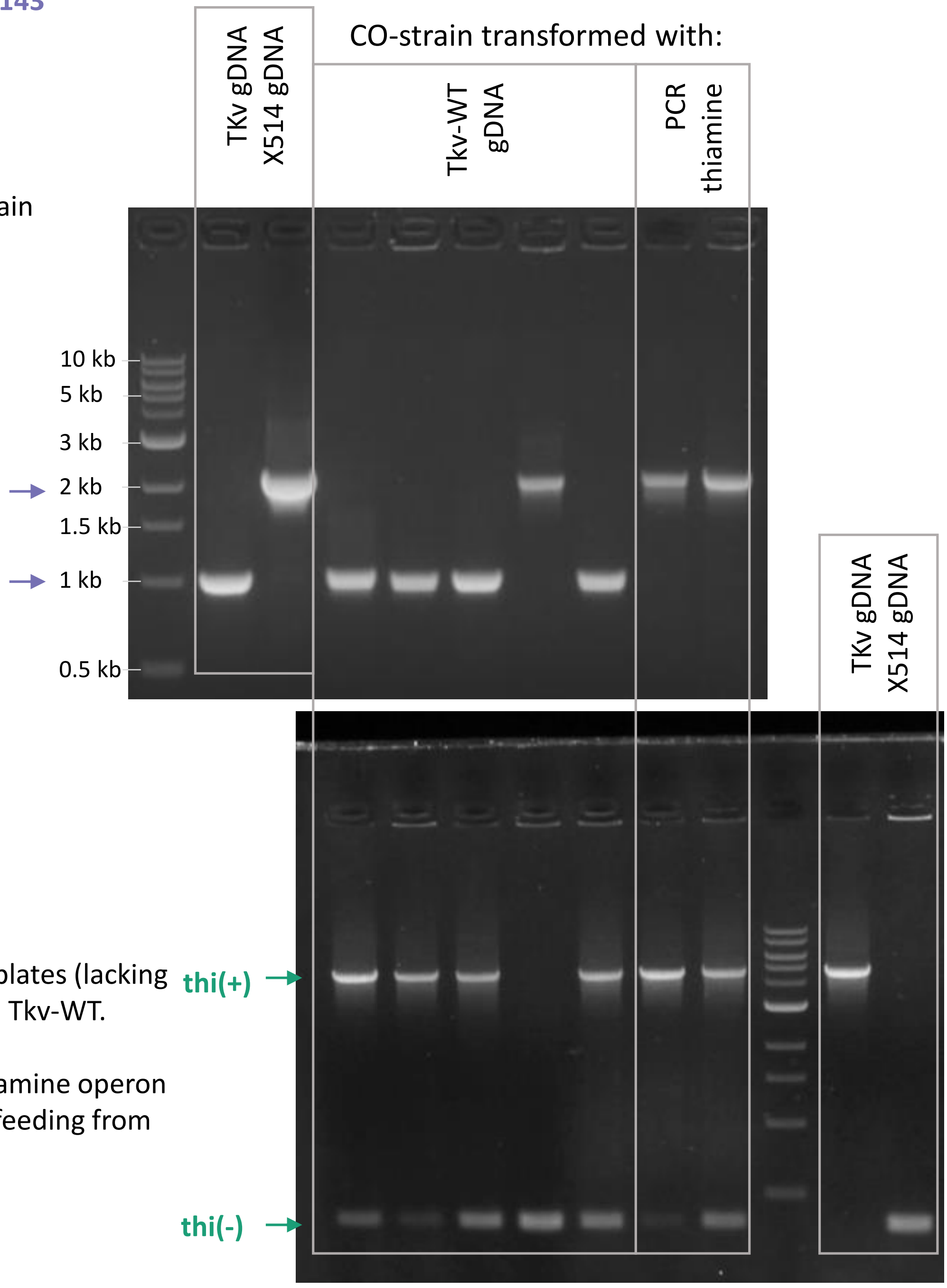

**Figure S4**: Location and contents of GC-rich islands in *T. kivui* genome
(A) Visualization of *T. kivui* genome GC content, with the three regions exceeding 50% GC numbered. (B) Table showing the position and GC content of each region (left) and gene contents of the three GC-rich islands (right).

**A)**

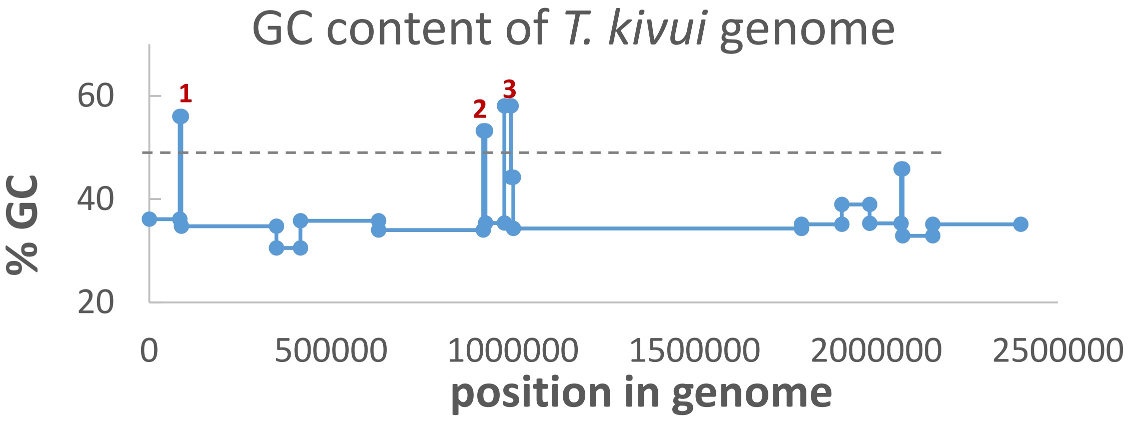


**B)**

| Start (bp) | Stop (bp) | Length (bp) | GC (%) | |
|---|---|---|---|---|
| 1 | 83798 | 83798 | 36.12 | |
| 83799 | 88779 | 4981 | 56.03 | Island1 |
| 88780 | 350232 | 261453 | 34.73 | |
| 350233 | 416054 | 65822 | 30.53 | |
| 416055 | 631246 | 215192 | 35.79 | |
| 631247 | 919081 | 287835 | 34.02 | |
| 919082 | 925597 | 6516 | 53.24 | Island2 |
| 925598 | 977227 | 51630 | 35.34 | |
| 977228 | 995977 | 18750 | 58.06 | Island3 |
| 995978 | 1001804 | 5827 | 44.24 | |
| 1001805 | 1794776 | 792972 | 34.29 | |
| 1794777 | 1905340 | 110564 | 35.11 | |
| 1905341 | 1982833 | 77493 | 38.97 | |
| 1982834 | 2068932 | 86099 | 35.33 | |
| 2068933 | 2072880 | 3948 | 45.87 | |
| 2072881 | 2155516 | 82636 | 32.92 | |
| 2155517 | 2397824 | 242308 | 35.11 | |

| ORF | Product |
|---|---|
| TKV_RS00400 | 16S ribosomal RNA |
| TKV_RS00405 | 23S ribosomal RNA |
| TKV_RS00410 | 5S ribosomal RNA |

| ORF | Product |
|---|---|
| TKV_RS04655 | cobaltochelatase subunit CobN |
| TKV_RS04660 | energy-coupling ABC transporter permease |
| TKV_RS04665 | PDGLE domain-containing protein |
| TKV_RS04675 | HD domain-containing protein |
| TKV_RS04680 | diguanylate cyclase |

| ORF | Product |
|---|---|
| TKV_RS04920 | ABC transporter permease |
| TKV_RS04925 | nickel ABC transporter permease subunit NikC |
| TKV_RS04930 | ABC transporter ATP-binding protein |
| TKV_RS04935 | ABC transporter ATP-binding protein |
| TKV_RS04940 | hypothetical protein |
| TKV_RS04945 | methyltransferase domain-containing protein |
| TKV_RS04950 | ABC transporter substrate-binding protein |
| TKV_RS04955 | cobaltochelatase subunit CobN |
| TKV_RS04960 | hypothetical protein |
| TKV_RS04965 | cobaltochelatase subunit CobN |
| TKV_RS04970 | nickel ABC transporter permease subunit NikC |
| TKV_RS13245 | ATP-binding cassette domain-containing protein |

**Figure S5**: Growth curves – all replicates

(A) Growth of *T. kivui* WT (orange) and CO-strain (blue) on glucose in NV medium (open shapes) or in NV medium with added thiamine (gray circles). Shown are both replicates

(B) complex medium with 10 mM glucose (black shapes) or 5 mM trehalose (gray shapes), shown are both replicates

(C) 20 g/L yeast extract as source of trehalose – optical density (WT = circles, CO strain = triangles), and trehalose concentration (squares with dashed lines). Shown are technical duplicates of biological replicates, with a single trehalose timecourse. Start and 9 hour measurements of trehalose concentrations are shown for all four samples, with error bars. Time zero trehalose measurements are slightly shifted on the X-axis for easier visibility

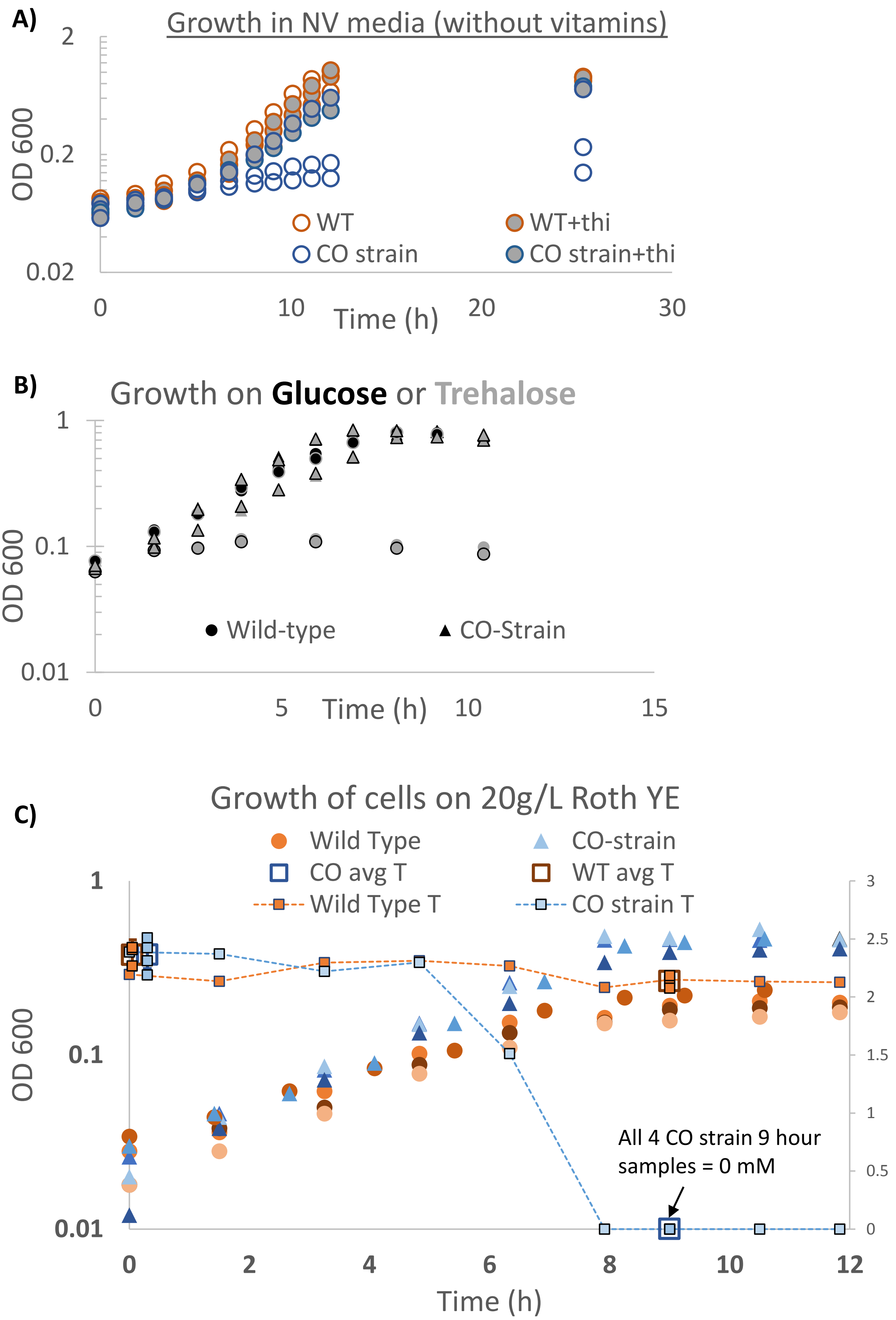

Supplement: Supplementary file 1 — Supplementary Material [file 43705_2022_211_MOESM1_ESM.pdf]
